# Visualization of membrane-stabilized SorCS2 *cis* interactions

**DOI:** 10.1016/j.yjsbx.2026.100155

**Published:** 2026-07-29

**Authors:** J. Wouter Beugelink, Bert J.C. Janssen

**Affiliations:** Structural Biochemistry, Bijvoet Centre for Biomolecular Research, Faculty of Science, Utrecht University, Universiteitsweg 99, 3584 CG Utrecht, The Netherlands

**Keywords:** SorCS2, subtomogram averaging, cryo-ET, proteoliposomes

## Abstract

Members of the Vps10p receptor family regulate protein trafficking and cellular differentiation in the nervous system. Previous structural studies of the dimeric Vps10p family member SorCS2 have focused on isolated ectodomains, revealing substantial structural plasticity but overlooking the influence of the membrane association on receptor organization. Here we establish two complementary tools for reconstituting the SorCS2 ectodomain on proteoliposomes in its native orientation: non-covalent coupling via a C-terminal His-tag and nickel affinity, and covalent attachment via strain-promoted alkyne–azide cycloaddition using a C-terminal azide. We visualize the SorCS2 membrane-associated protein organization using electron cryo-tomography and obtain a nanometer resolution subtomogram average of the His-tag coupled SorCS2 ectodomain dimer. Four distinct, previously unreported, SorCS2 dimer-of-dimer arrangements are observed. The two most prominent interactions form through “head-to-side” docking of a Vps10p domain to the Vps10p and PKD core of another dimer, and “head-to-head” symmetric interactions between the Vps10p and SoMP domains of two dimers. Two less frequent assemblies comprise “side-by-side” interactions between the beta-propeller and 10CC domains and symmetrical “face-to-face” beta-propeller top face interactions. Together these interactions organize SorCS2 into two distinct helical arrangements and small receptor clusters on liposome surfaces. The promiscuity of membrane-stabilized SorCS2 *cis* interactions supports a more general mechanism in which the organization of receptor systems is influenced by membrane association. The tools presented here provide a versatile platform for visualizing ectodomain-mediated receptor assemblies in a membrane context.

## Highlights

• Proteoliposomes were generated using two different methods.
• The structure of SorCS2 on a membrane was determined using subtomogram averaging.
• SorCS2 forms a helical arrangement on tubular liposomes.
• Four novel SorCS2 dimer-of-dimer *cis* arrangements were found.
• The organization of receptor systems may be influenced by membrane association.

## Graphical abstract

**Figure:**
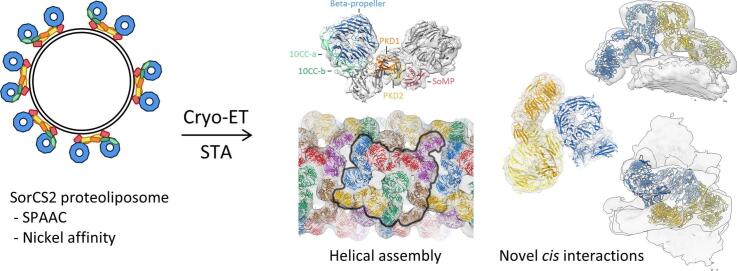

## 1 Introduction

Sortilin, SorLA, and SorCS1–3, are the five members of the Vps10p family (vacuolar protein sorting 10) of receptors that together with neurotrophin ligands and receptors regulate growth and differentiation in the developing and mature nervous system (Malik and Willnow, 2020). Described mechanisms of Vps10p family members are twofold: they control the subcellular localization of protein partners through trafficking (Vaegter et al., 2010; Savas et al., 2015; Ma et al., 2017; Malik et al., 2019), secretion (Chen et al., 2005), and endocytosis (Jacobsen et al., 2001; Hermey et al., 2003), and they act as (co-)receptors in signaling pathways that are activated by extracellular cues (Nykjaer et al., 2004; Deinhardt et al., 2011; Glerup et al., 2014; Glerup et al., 2016). SorCS2 acts in both of these manners in different contexts. In motor neurons, SorCS2 binds the growth factor progranulin (PGRN) intracellularly, and regulates its trafficking and secretion, while simultaneously being a required receptor for neurotrophic signaling by macrophage-released PGRN (Thomasen et al., 2023). SorCS2 forms a ternary complex with the neurotrophin receptor p75^NTR^ and the neurotrophin pro-nerve growth factor (proNGF) that induces acute growth cone collapse in neurites (Deinhardt et al., 2011; Glerup et al., 2014). The SorCS2 ectodomain has a direct interaction with p75^NTR^, and they cooperatively bind neurotrophins (Glerup et al., 2014). Similarly, SorCS2 binds the neurotrophin receptor TrkB to facilitate pro-brain-derived neurotrophic factor (proBDNF) signaling in an activity-dependent manner (Glerup et al., 2016). As such, SorCS2 is an important regulator of growth cones of developing neurons, and is linked to neurodevelopmental disorders (Olsen et al., 2021).

Vps10p family members are type I transmembrane proteins, consisting of multi-domain extracellular segments, and cytoplasmic tails for signaling and adaptor binding. Structurally, Vps10p family members are characterized by their signature Vps10p domain, which itself consists of three domains, a ten-bladed beta-propeller and two small cysteine-rich domains (10CC-a and 10CC-b) that wrap onto the bottom face of the beta-propeller (Quistgaard et al., 2009). While the Sortilin ectodomain only contains the Vps10p part, the ectodomains of SorCS1–3 are bigger, also including two PKD domains, and a unique SorCS membrane-proximal domain (SoMP) (Leloup et al., 2018). Dimerization is described for all Vps10p family members. Sortilin dimerization is regulated by pH and causes release of cargo in late endosomes in its function as a trafficking receptor (Leloup et al., 2017; Januliene et al., 2017a). The extracellular portions of SorCS1–3 are constitutive dimers in solution, with a symmetrical core formed by cross-braced PKD domains (Leloup et al., 2018). SorCS dimerization is not dynamic, yet it might be regulated by glycosylation state, as more dimers are observed in solution upon deglycosylation of the ectodomains (Januliene et al., 2017b). In solution, structural plasticity of SorCS dimers can be inferred from the distinct conformations, that are either compact or extended, observed in crystal structures and electron microscopy studies (Leloup et al., 2018; Dong et al., 2022).

As is the case for Vps10p family members, the structural study of membrane-bound receptors, signaling molecules, and adhesion complexes is often done through crystallization or single particle cryo-EM studies on isolated ectodomains, forgoing the context of the membrane (Quistgaard et al., 2009; Leloup et al., 2018; Leloup et al., 2017; Dong et al., 2022; Kitago et al., 2015; Zhang et al., 2022). However, attachment to the membrane has biological functions. Some proteins enable communication through the cell membrane. Others have roles in cellular adhesion, constraining the distances between cells, or giving rise to specificity between cellular connections, shaping cells into tissues (Honig and Shapiro, 2020). The context of the membrane and steric effects that it brings can be important for the architecture of signaling and adhesion complexes. Conformations that can potentially be adopted in solution, are disallowed or unfavorable due to the steric presence of a membrane, or may be stabilized by direct interactions with the membrane (Gurdap et al., 2022). Interactions on the same surface, in *cis*, and on opposing surfaces, in *trans*, rely on the membrane to provide a surface to associate on. Such interactions are potentially weak in solution, impeding their structural characterization, but could still be physiologically relevant (Wright, 2009). Membranes reduce dimensionality of diffusion and increase effective local concentrations (Minton, 1995; Mishra and Johnson, 2021). Proteins can be actively targeted to clusters on the membrane, such as in lipid rafts or tetraspanins-enriched microdomains (Simons and Ikonen, 1997; Yáñez-Mó et al., 2009). *Cis* interactions can be of low affinity in solution, but still be relevant due to this local concentration effect (Wu et al., 2010). *Trans* interactions also benefit from increased local concentration, and gain avidity effects by being on opposing surfaces (Aricescu and Jones, 2007).

Ideally, one would investigate the structure of a protein as it functions in the cell. Advances in electron microscopy, thinning of vitrified samples, and data processing, have enabled the *in situ* electron cryo-tomography study of membrane-bound proteins in their native environment (Briggs, 2013; Nogales and Mahamid, 2024; Majumder and Zhang, 2025). However, this is most effectively applied to targets that are large, abundant, and/or filamentous (Majumder and Zhang, 2025; Turk and Baumeister, 2020). Many signaling receptor–ligand interactions and adhesive processes are too elusive to be captured in a native state, due to their limited size or low abundance. Thus, adding back the context of the membranes in model systems can bridge the gap between membrane-less study of ectodomains and *in situ* tomography. Such systems can be derived from natural and artificial membranes. When overexpressed recombinantly, membrane proteins can accumulate and be secreted on extracellular vesicles spontaneously, or specifics tags can effectively target proteins to secreted vesicles (Zeev-Ben-Mordehai et al., 2014a; Gonzalez-Magaldi et al., 2025). Such vesicles have been isolated and imaged successfully, revealing the architecture of ectodomains in context of the membrane. Other approaches rely on artificial membranes, liposomes functionalized with lipids that bear affinity to protein tags (He et al., 2009; Brasch et al., 2019). This maintains some of the advantages of working with isolated ectodomains, including more options to optimize recombinant expression for yield and stability, and allows for control over the membrane composition.

Here, we investigate the application of coupling proteins to liposomes for cryo-TEM imaging. Two tools, a system based on strain-promoted alkyne–azide cycloaddition (SPAAC) for generation of specific covalent proteoliposomes and a system based on histidine-tag affinity to nickel ions allowed for imaging of several membrane-attached proteins. As the histidine-based method resulted in more efficient membrane coupling, we used this method to detail the *cis* interactions that SorCS2 can have on a membrane surface. Through subtomogram averaging, a nanometer resolution density map of deglycosylated SorCS2 bound to a membrane was obtained, resembling the more compact NGF-bound conformation (Leloup et al., 2018). In the tomograms helical arrangements of SorCS2 dimers were found, formed by *cis* interactions on tubular membranes. The most prominent interactions form through “head-to-side” docking of a Vps10p domain to the Vps10p and PKD core of another dimer, and “head-to-head” symmetric interactions between the Vps10p and SoMP domains of two dimers, as well as a minor “side-by-side” interaction. By mapping back particle orientations to the tomogram frame, we found that such *cis* interactions also form on non-tubular liposomes. We next added PGRN or p75^NTR^-NGF to glycosylated SorCS2 in our workflow to collect two more datasets. Independent analysis of these datasets did not lead to resolving the heteromeric complexes, but instead reproduced the “head-to-head” dimer-of-dimer interface and additionally a fourth “face-to-face” *cis* interaction mode of SorCS2 dimers on the membrane. In total we identify four distinct SorCS2 dimer-of-dimer *cis* interactions, none of which were previously reported. The work shows that the membrane association helps in stabilizing *cis* interactions of the extracellular portion of SorCS2 and suggests that the role of the membrane could be relevant for *cis* interactions of other transmembrane receptors and adhesion systems.

## 2 Results

### 2.1 Two tools for coupling proteins to liposomes

**Fig. 1:**
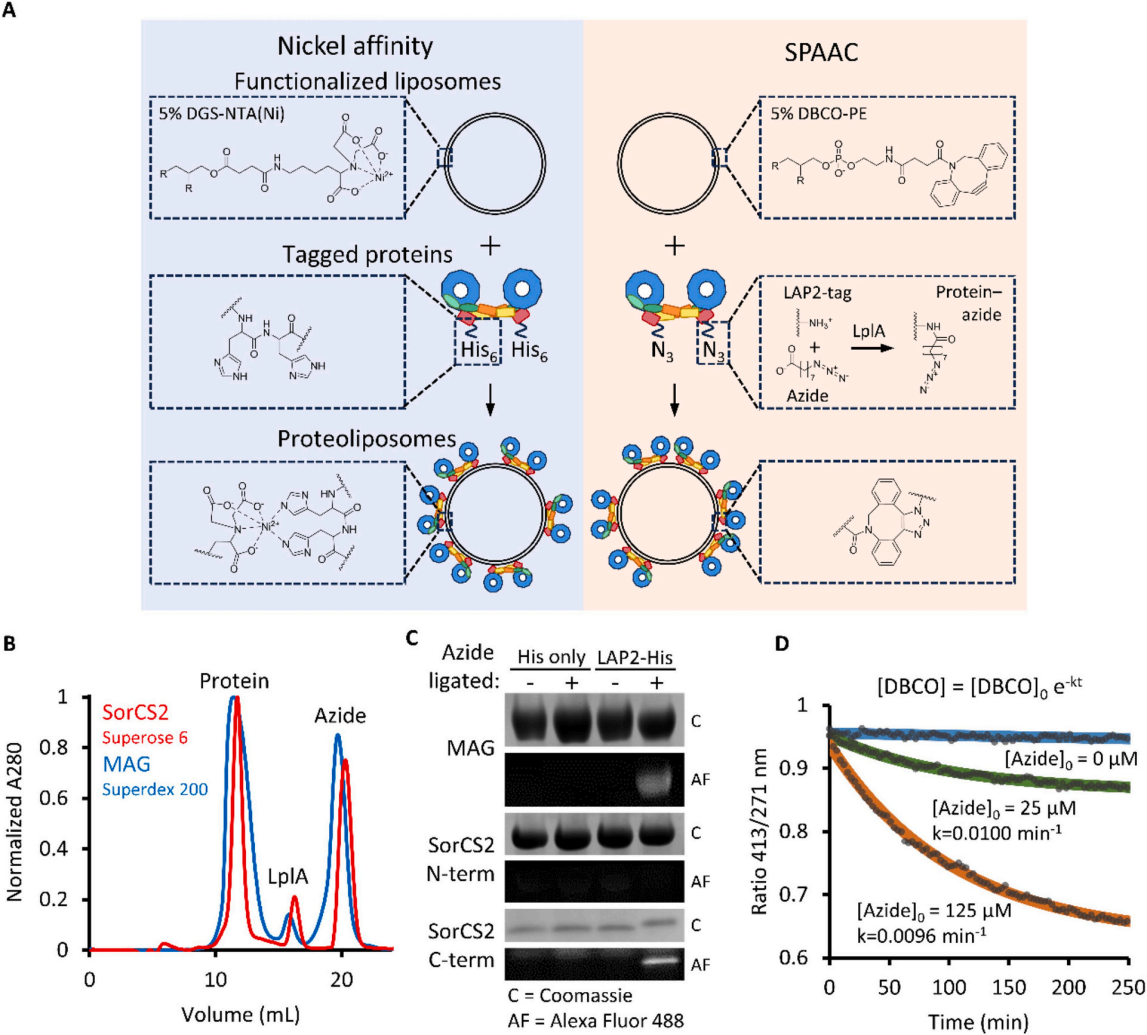
Preparation of proteoliposomes. A) Overview of two tools for generating proteoliposomes: Nickel affinity and strain-promoted alkyne–azide cycloaddition. B) Re-purification of proteins after azide ligation. In blue, the MAG ectodomain on a Superdex 200 column. In red, the SorCS2 ectodomain on a Superose 6 column. The ectodomains are efficiently separated from the LplA enzyme and unbound excess azide. C) SDS-PAGE of unligated and azide-ligated MAG and SorCS2 ectodomain with or without the LAP2 tag, incubated with DBCO–Alexa Fluor 488 conjugate. Fluorescent signal indicates covalent coupling through SPAAC, and is only observed with azide ligation to the LAP2 tag, indicating specificity of the azide ligation. Full gel in Supplementary fig. 1. D) Ratio of 413 nm to 271 nm absorbance to follow the click reaction of 8-azidooctanoic acid with DBCO-PE containing liposomes. Active DBCO is present on liposome surfaces, as indicated by the reduction in absorbance over time in the presence of azide. A pseudo-first-order kinetic model was fitted to the data. (For interpretation of the references to colour in this figure legend, the reader is referred to the web version of this article.)

Two methods for coupling protein ectodomains to liposomes were considered: through affinity of hexahistidine-tags (His-tag) to immobilized nickel ions, and through strain-promoted alkyne–azide cycloaddition (SPAAC; Fig. 1a). Liposomes are functionalized by including 5% of either the nickel salt of nitrilotriacetic acid coupled to 1,2-dioleoyl-sn-glycero-3-succinate (DGS-NTA(Ni)) or dibenzocyclooctyne-phosphatidylethanolamine (DBCO-PE). Purified extracellular segments of the proteins have a His-tag and, optionally, also a lipoic acid acceptor peptide tag (LAP2 tag) (Puthenveetil et al., 2009) incorporated at the membrane attachment site, e.g. C-terminally for type I transmembrane proteins. His-tagged proteins can be coupled to NTA(Ni) functionalized liposomes. LAP2-tagged proteins are enzymatically labelled, at the lysine in the tag, with a lipoic acid analog, 8-azidooctanoic acid (hereafter referred to as ‘azide’), by a variant of the *E. coli* enzyme lipoic acid ligase (LplA^W37V^) (Baruah et al., 2008; Uttamapinant et al., 2013). The azide-containing protein is covalently coupled to the DBCO-functionalized liposomes through SPAAC. Both methods result in coupling to the membrane with relatively short linkers to best mimic physiological membrane attachment in a native topology. Coupling through nickel affinity is non-covalent and can result in intermixing of proteins between liposomes. Coupling through SPAAC is covalent and results in distinct liposome populations that cannot intermix, a requirement for studying heterophilic *trans* interactions.

### 2.2 Generation of covalent proteoliposomes using a strain-promoted click reaction

Covalent coupling of protein ectodomains to liposomes was accomplished through SPAAC. Azide ligation to the LAP2-tag was performed with the ectodomain of SorCS2, and as an extra control, the ectodomain of myelin-associated glycoprotein (MAG). The azide-ligated proteins could be efficiently separated from the lipoic acid ligase enzyme and unreacted azide using size-exclusion chromatography (Fig. 1b). The efficacy and specificity of azide ligation, as well as its reactivity to DBCO, was tested by incubating ectodomains with a DBCO–Alexa Fluor 488 conjugate (Fig. 1c, Supplementary fig. 1). Fluorescence is detected for LAP2-tagged, azide-ligated MAG, but not in controls that either lack the LAP2-tag or azide ligation, indicating specific labelling of the LAP2-tag. SorCS2 runs on the gel in two bands, at approximately 104 kDa and 10 kDa (Supplementary fig. 1), which we assume to correspond to the two-chain cleaved variant found in glial cells (Glerup et al., 2014). Indeed, the C-terminal part of LAP2-tagged, azide-ligated SorCS2 is fluorescently labelled, indicating specific azide ligation to the LAP2-tag. Similarly to MAG, fluorescence is detected only in presence of the LAP2-tag, and only after azide ligation, confirming that the azide is coupled specifically onto the tag. Next, liposomes with reactive DBCO on the surface were made, by incorporating 5% DBCO-PE. As DBCO is a chromophore, and this property is lost upon reacting with an azide, the click reaction can be followed by measuring absorbance. Over time DBCO-incorporated liposomes at 125 μM DBCO concentration, give stable signal in absence of azide, and react in presence of 25 or 125 μM azide, resulting in lowering of the signal (Fig. 1d). While SPAAC reactions in solution are usually described as second-order reactions (Luu et al., 2024; Pringle and Knight, 2025), in our setup on liposomes, the data are more accurately modeled as following first-order kinetics. This indicates that the reaction is limited by diffusion, similar to SPAAC reactions on polymer brushes (Orski et al., 2012). While the final amount of reacted DBCO is dependent on the azide concentration, the rate at which it reacts is not, with the rate constant being approximately 0.01 min^−1^ at initial azide concentrations of 25 μM or 125 μM. Using the above-described workflow, azide-labeled MAG and SorCS2 were covalently coupled to DBCO-containing liposomes through SPAAC.

### 2.3 Electron cryo-tomography imaging of proteins coupled to liposomes

**Fig. 2:**
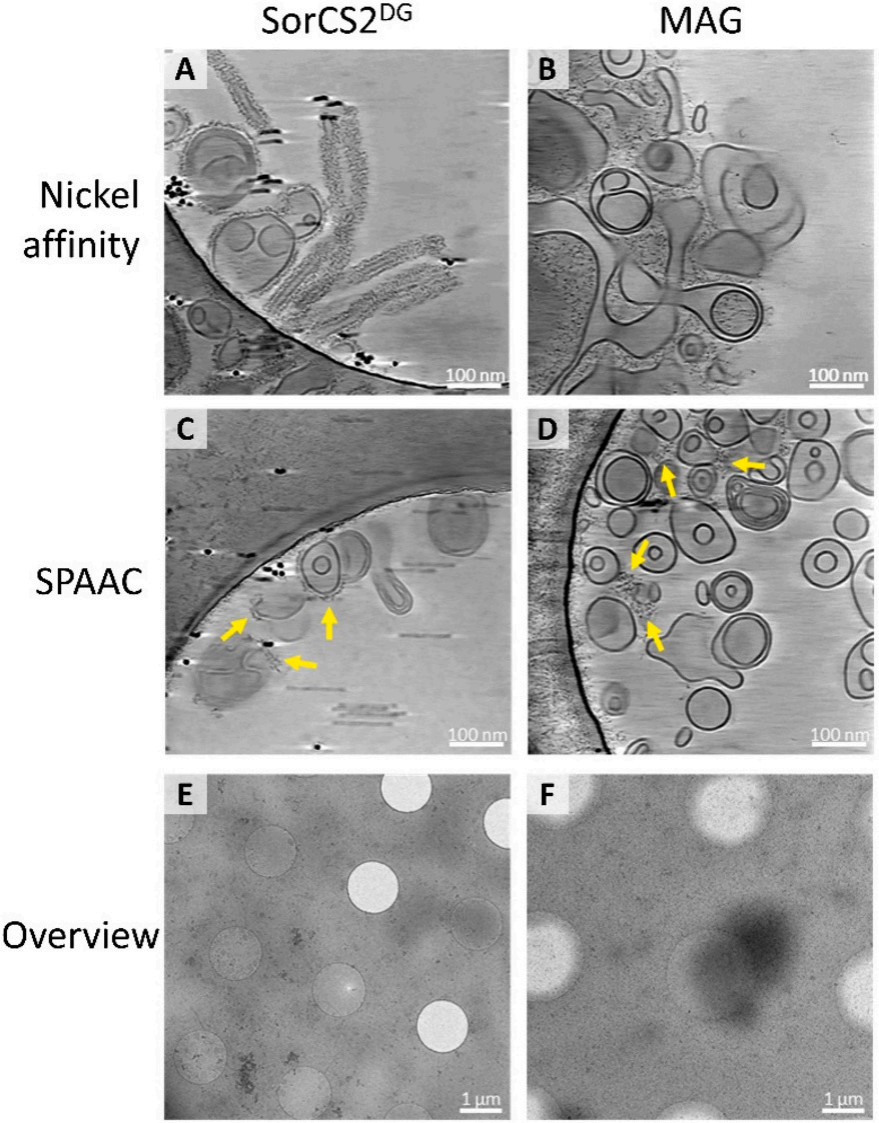
Imaging of proteoliposomes in the transmission electron microscope. A) Slice through a denoised tomogram of proteoliposomes made of NTA(Ni)-functionalized liposomes and the SorCS2 ectodomain. Protein density is observed as “bristle” on membrane surfaces. Decorated membranous tubules are also observed. B) Slice through a denoised tomogram of proteoliposomes made of NTA(Ni)-functionalized liposomes and the MAG ectodomain. Protein density is observed as “speckled” between membrane surfaces. C) Slice through a denoised tomogram of proteoliposomes made of DBCO-functionalized liposomes and the SorCS2 ectodomain. Yellow arrows indicate bound protein. D) Slice through a denoised tomogram of proteoliposomes made of DBCO-functionalized liposomes and the MAG ectodomain. Yellow arrows indicate bound protein. E) Lower magnification micrograph of the NTA(Ni)–SorCS2 proteoliposomes, which are distributed evenly on the grid. F) Lower magnification micrograph of the NTA(Ni)–MAG proteoliposomes. A large cluster of liposomes, several microns in diameter, is observed. (For interpretation of the references to colour in this figure legend, the reader is referred to the web version of this article.)

In order to confirm conjugation of proteins to functionalized liposomes, either through SPAAC or histidine-nickel affinity, tilt series were collected in a transmission electron microscope under cryogenic conditions. The ectodomains of MAG and Endo Hf deglycosylated SorCS2 (SorCS2^DG^) were conjugated to liposomes using both methods. Samples containing liposomes conjugated with MAG, but not with SorCS2^DG^, displayed aggregates that were visible by eye, and were vortexed to break up liposome clusters before applying to EM grids. In all four samples, protein density associated with liposomes was observed in the reconstructed tomograms (Fig. 2a-d). In the MAG samples, liposomes were found on the grids in micrometer-sized clusters (Fig. 2e), while no clusters were observed in SorCS2^DG^ samples (Fig. 2f). Correspondingly, protein density is present on SorCS2^DG^-conjugated liposomes in a “bristle” morphology along the membrane, while protein density is present in a “speckled” morphology between the membranes of MAG-conjugated liposomes, suggesting that MAG mediates *trans* interactions between liposomes. For both proteins, the coverage of conjugated proteins on the membrane was much higher for samples prepared using the nickel affinity method, as compared to SPAAC. Also, apparent distortions in membrane shapes, deviating from typical round shapes of liposomes resulting in elongated and tubulated membranes, were more frequent in nickel affinity samples, potentially caused by the higher degree of membrane coverage. In the partially decorated SPAAC liposomes, protein localizes to patches on the surface, and seems unevenly distributed between liposomes, in that some liposomes are fully decorated, while others are not decorated at all. Potentially, DBCO-PE lipids are not homogeneously distributed between liposomes, explaining the patchy protein distribution. Both methods enable the conjugation of proteins to liposomes, but the coverage and distribution of conjugated proteins is more optimal for the nickel affinity-based method, which was therefore selected for further experiments.

### 2.4 Subtomogram averaging of the SorCS2 ectodomain on a membrane

**Fig. 3:**
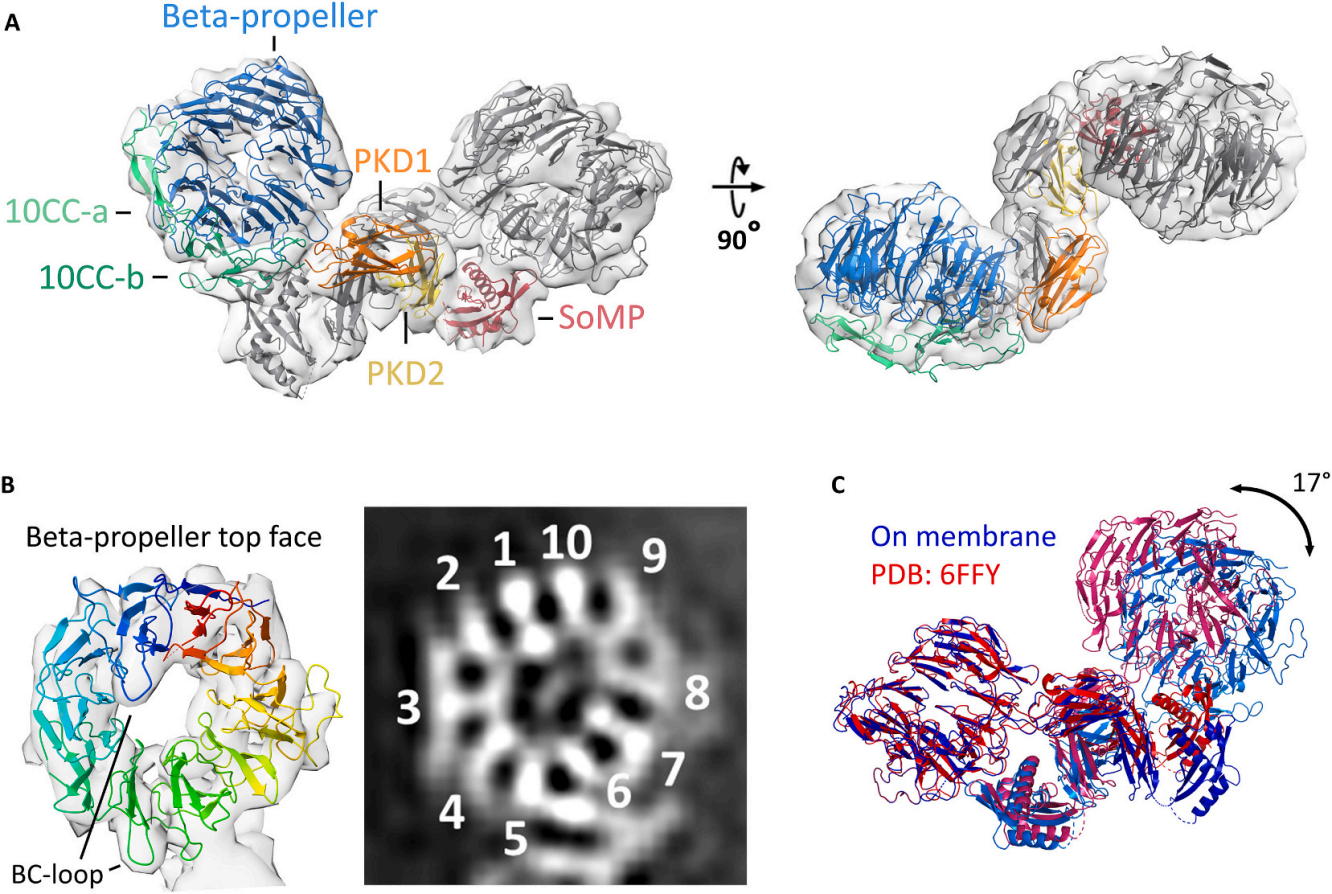
Subtomogram average of SorCS2^DG^ on a membrane. A) Overall architecture of the SorCS2 domain, colored by domain for a single protomer. This model was obtained by rigid-body refinement of the crystal structure of NGF-bound SorCS2 (PDB:6FFY) to the map (Leloup et al., 2018). B) All ten beta-blades of the beta-propeller are clearly visible, and the BC-loop of the first and fifth blade fit the density well, indicating good map quality. C) Comparison of the structure of SorCS2 on a membrane to NGF-bound SorCS2 (PDB:6FFY) (Leloup et al., 2018). The structures are aligned on one beta-propeller, and deviations in the position of the other beta-propeller are indicated.

The structure of the SorCS2^DG^ full ectodomain, coupled through histidine–nickel affinity on a membrane, was determined to nanometer resolution. First, positions were picked by oversampling the surface of tubular and rounded vesicles (Supplementary fig. 2a). The initial set of 129 thousand oversampled positions was curated down to a set of 16,222 unique particle positions through classifications and by removing overlaps resulting from translational sampling. This set of particles was then refined to a final resolution of 11 Å (Supplementary fig. 2b). A second set of particles was selected using a template matching procedure, which after several rounds of classification resulted in a set of 13,189 particles which refined to 9.6 Å resolution (Supplementary fig. 2c). Of the total 29,411 positions identified using the two methods, only 3798 were identified by both methods, leaving 25,614 unique particles. The Fourier shell correlation between averages of the unique particles for each dataset extended to 11 Å, indicating that both methods converged to the same structural conformation (Supplementary fig. 2c). Further processing resulted in a final map of membrane-bound SorCS2^DG^ at a resolution of 10 Å (Fig. 3a).

In the final refined map, expected features such as the ten blades of the beta-propeller domain and the extended BC loops of the first and fifth blades, are readily visible in the density, supporting the 10 Å resolution (Fig. 3b). The membrane-bound SorCS2^DG^ adopts a conformation that best resembles the more compact conformation found in the NGF-bound crystal structure of glycosylated SorCS2 (PDB:6FFY) (Leloup et al., 2018). Refinement of this atomic model against the density reveals some changes in domain orientations, as compared to the crystal structure (Fig. 3c). When aligned on one Vps10p domain, the other Vps10p domain displaces by a hinge of 17°. This also brings the C-termini closer together (75 Å in the crystal structure versus 69 Å in the membrane-associated conformation), which possibly accommodates stronger membrane curvature.

### 2.5 Cis interactions of membrane-bound SorCS2^DG^

**Fig. 4:**
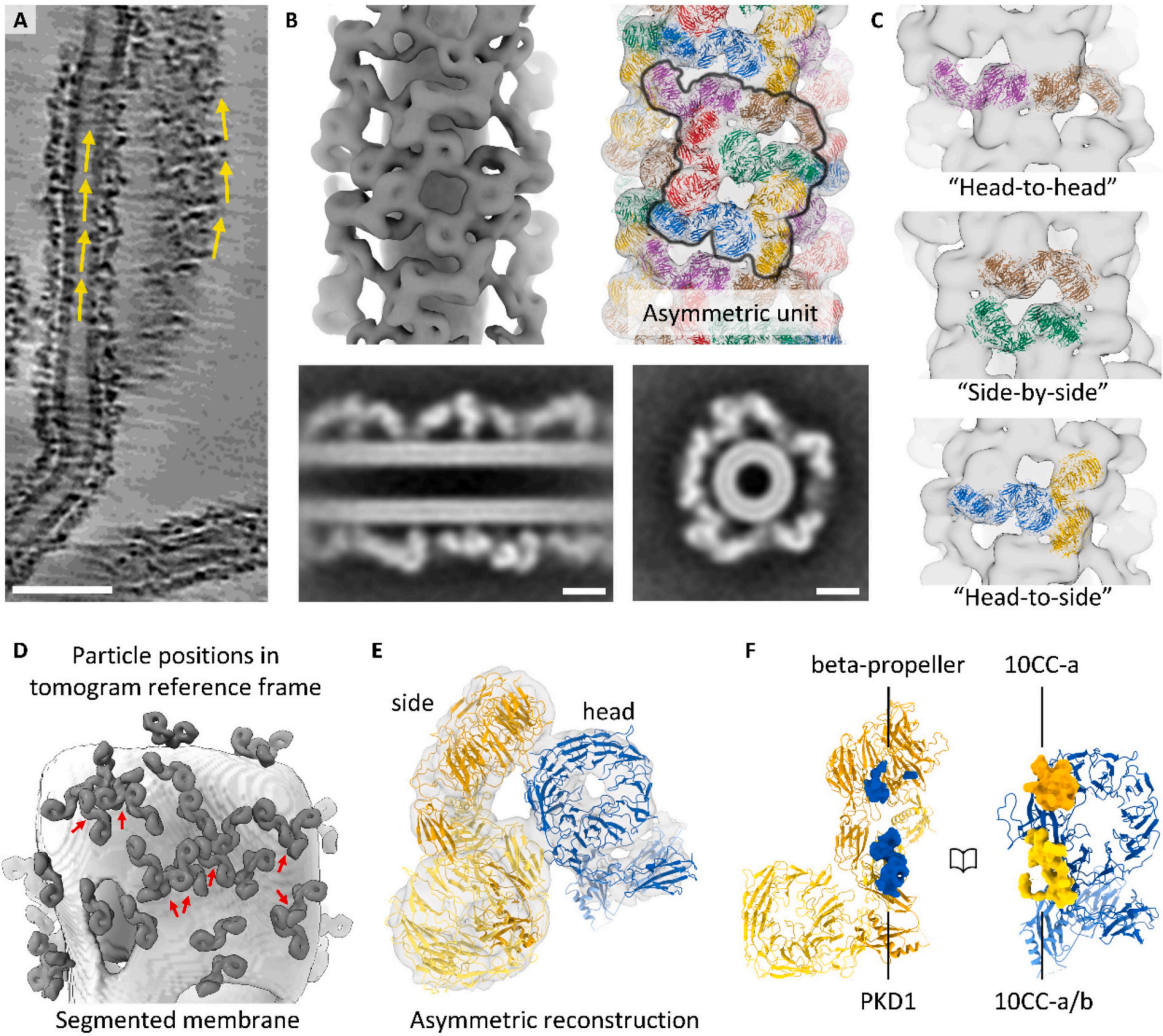
*Cis* interactions of SorCS2 dimers on a membrane. A) Slice through a denoised tomogram of SorCS2^DG^ proteoliposomes, showing tubular membranous structures, decorated with potentially periodic protein density, indicated by arrows. Scale bar is 50 nm. B) Helically refined protein density along tubular liposomes. A clear bilayer is visible in the subtomogram average. The proteinaceous density is completely explained by an asymmetric unit of six SorCS2 dimers, which are outlined, and each helically equivalent dimer is colored the same. Scale bars are 100 Å. C) Isolated views of the three interfaces forming the helical assembly, with SorCS2 dimers colored as in B). D) Segmentation of a membrane and SorCS2^DG^ particle poses plotted back to the tomogram reference frame. The SorCS2^DG^ density map was filtered to 20 Å resolution for clarity. *Cis* interactions, specifically the head-to-side interaction, is also found on spherical liposomes. E) Asymmetric reconstruction of the head-to-side interaction with rigid-body refined models. F) Open book representation of the “head-to-side” interaction. The bottom face of the beta-propeller of one protomer interacts with the 10CC-a domain of the docked dimer, while the PKD1 of the other protomer interacts simultaneously with the 10CC-a and 10CC-b of the docked dimer. Residues within 7 Å of the interacting protomer are displayed as surface representation.

In reconstructions of the SorCS2^DG^ dimer additional densities were present, hinting at higher-order organization. Furthermore, on the pervasively present tubular membranes, repetitive features can be observed along the tubular axis (Fig. 4a). This was investigated by picking positions along the hollow cores of the membranous tubules and extracting particles with a box size that contains the entire diameter of the tubule. Classifications and refinement of these particles showed that SorCS2 can be organized on tubular membranes in a triple-start helical assembly (Fig. 4b). The helical parameters were refined to a rise of 32.6 Å and a twist of −134.4 degrees. An asymmetric unit consisting of six distinct SorCS2 dimers can fully explain the proteinaceous density. Four out of the six dimers are arranged in a pseudo-four-fold arrangement, with two more dimers bridging between neighboring four-fold arrangements.

Three distinct interfaces between SorCS2 dimers are observed. Vps10p domains of adjacent dimers are interacting either symmetrically in a “head-to-head” or “side-by-side” arrangement, or four times more frequently in an asymmetric “head-to-side” mode, with the Vps10p domain of a dimer docking in between the Vps10p domain and PKD core of another dimer (Fig. 4c). A rigid helical assembly would restrain the membrane to a straight tube with a uniform circular cross-section and diameter of 135 Å, yet in the tomograms it is readily apparent that the tubules are neither straight, nor have a consistent diameter (Fig. 2a, Fig. 4a). Classifications of the helically refined particles without realignment reveal many defects in the assembly, indicating that the symmetry is indeed not strictly followed, but present in patches along tubes. When mapping back the 25,614 refined particle poses that provided the 10 Å resolution map to the tomographic reference frame, not only particles along membranous tubules, but also those on round vesicles, seem to form *cis* interactions (Fig. 4d). Subjecting SorCS2 dimer particles on non-tubulated liposomes to nearest-neighbor analysis also indicates the presence of *cis* interactions in this set (Supplementary fig. 2d). Most often, one dimer hosts the Vps10p domain of another between its Vsp10p domain and PKD core, resembling the “head-to-side” interaction found on the membrane tubules. In order to better describe this interaction, the set of 25,614 SorCS2^DG^ particles was classified and refined with a wider mask and symmetry relaxation, resulting in a 12 Å map from 7398 particles with a third Vps10 domain clearly defined (Fig. 4e). The top face beta-propeller domain of one protomer of the host dimer interacts with the 10CC-a segment of the docked dimer, most likely involving the BC loops of the first and tenth blade. The first PKD domain of the other protomer interacts with the 10CC-a and 10CC-b domains of the docked dimer. Out of the three distinct *cis* dimers, this “head-to-side” 10CC-a/10CC-b–beta-propeller/PKD interaction, seems to be the most favorable.

### 2.6 Membrane-bound SorCS2^glyc^ also forms dimer-of-dimers

**Fig. 5:**
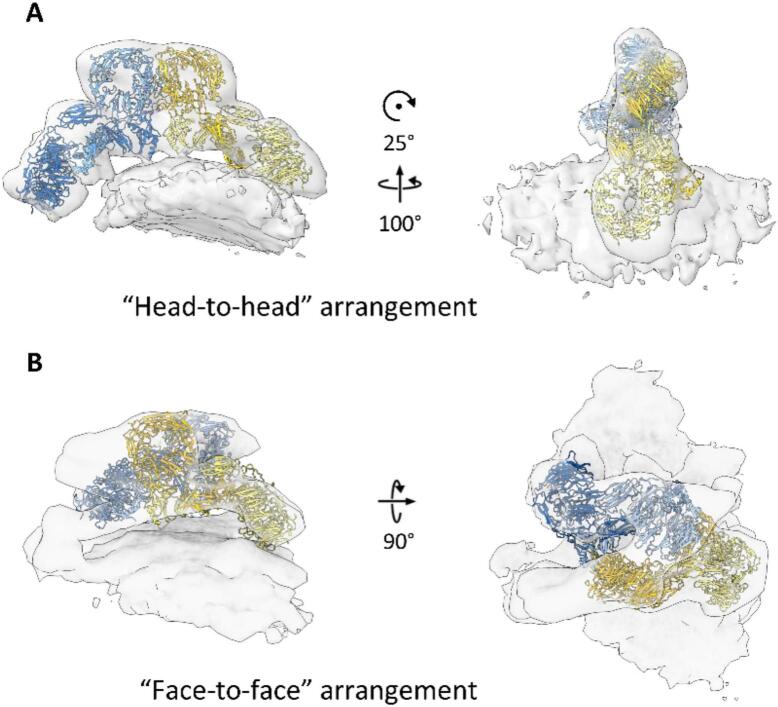
SorCS2 dimer-of-dimers on a membrane in the SorCS2(p75^NTR^/NGF) sample. Subtomogram averages of dimer-of-dimers in A) the “head-to-head” arrangement and B) the “face-to-face” arrangement. SorCS2 dimers are placed and rigid-body refined.

Since a ternary complex between SorCS2, p75^NTR^, and NGF does not form in solution (Leloup et al., 2018), we attempted to form this complex on a membrane surface. We first incubated Ni-NTA liposomes with glycosylated and His-tagged SorCS2 (SorCS2^glyc^) and p75^NTR^, then added untagged NGF, and finally vitrified the sample after a second incubation period. The positions of 30,828 membrane-associated densities were manually selected from denoised tomograms (Supplementary fig. 3). These were then aligned to the membrane density through unrestrained refinement, so that they share a common reference frame. An initial density map was obtained using half of the total dataset. In this set heterogeneity was resolved through several rounds of classification with restrained alignments in RELION 5, until a density resembling a SorCS2 beta-propeller could be interpreted from a set of 1897 particles. This map was used to steer alignments and classification of the full dataset (Supplementary fig. 3). While no complexes containing p75^NTR^ or NGF were identified, two dimer-of-dimer arrangements of membrane-bound SorCS2 were resolved. The first arrangement resembles the “head-to-head” arrangement found in the helical assembly of SorCS2^DG^, and likely uses the same interface consisting of the side of the VPS10p domain and the SoMP (Fig. 5a). The second dimer-of-dimer arrangement in this dataset forms using a symmetric interface that covers the top face of the beta-propeller, that we call the “face-to-face” arrangement (Fig. 5b). Classified maps contain density for either both or only the “head-to-head” arrangement, indicating that this is the more frequently formed *cis* interaction. SorCS2 dimers in either arrangement adopt an extended conformation previously reported for the deglycosylated, unliganded crystal structure (PDB:6FG9) (Leloup et al., 2018).

The (pro)NGF binding site in SorCS2 is located at the beta-propeller top face (Leloup et al., 2018). In both membrane-bound SorCS2^glyc^ dimer-of-dimer arrangements, the SorCS2 protomers have asymmetric positions with respect to the membrane plane. One of the beta-propellers is upright, and the other flat and near to the membrane plane. These two beta-propeller–membrane orientations give them distinct profiles of accessibility, and obscures one (pro)NGF binding site in each protomer. In the “head-to-head” arrangement the upright protomer forms the symmetrical “head-to-head” Vps10/SoMP interface with the adjacent dimer, which leaves the (pro)NGF binding site on the beta-propeller available. At the available (pro)NGF binding sites in the “head-to-head” arrangement map, no additional density is present, indicating NGF did not associate with the SorCS2 that we could localize in the sample. In the “face-to-face” arrangement, the beta-propeller top face forms a symmetrical interface, further limiting the availability of (pro)NGF binding sites.

### 2.7 Nickel-induced helical arrangements of SorCS2

**Fig. 6:**
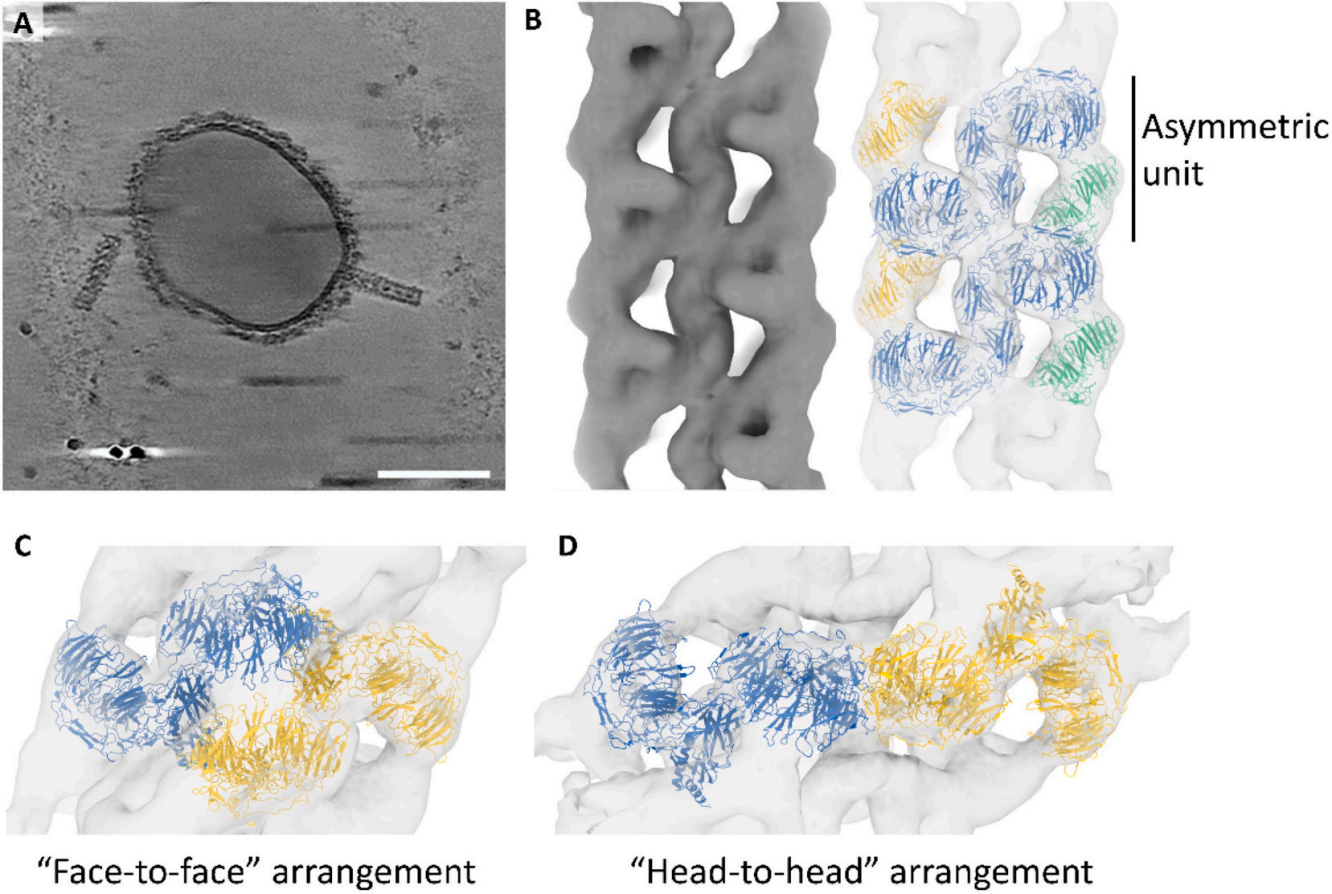
Nickel-stabilized helical arrangement in the SorCS2(PGRN) sample. A) Slice through a tomogram of NTA(Ni)–SorCS2 proteoliposomes with PGRN added. Liposomes are decorated, and small filaments are observed, both associated with the membrane and in solution. Slice thickness is 5 nm, scale bar is 100 nm. B) Subtomogram average of the tubules reveals that it consists of a helical assembly with an asymmetric unit of four SorCS2 dimers with D4 symmetry. C) Top view of the “face-to-face” arrangement that is found in this helical structure. D) Top view of the “head-to-head” arrangement, that is also found in the helical arrangement of SorCS2^DG^ on membranous tubules (see Fig. 4c).

In an attempt to determine the structure of the interaction between SorCS2 and PGRN, in-solution and membrane-bound complex samples were prepared for single-particle and tomographic analysis, respectively. Unfortunately, structure determination of a complex between SorCS2 and PGRN proved unsuccessful. Nonetheless the experiment did corroborate that glycosylated SorCS2 can adopt a previously observed extended conformation (Leloup et al., 2018) and form *cis* interactions. For these experiments, the PGRN protein sample was acquired commercially. Aggregates were visible in the single-particle sample, and extensive particle selection and 2D classification procedures did not produce a reconstruction of SorCS2, PGRN, or a complex between them. In the sample, long filamentous structures were observed, but those could not be reconstructed due to their association with aggregates (Supplementary fig. 4a). In the tomograms of membrane-bound SorCS2 incubated with PGRN, filamentous structures of similar dimensions were observed, both associated with membranes and in solution (Fig. 6a). Subtomogram averaging of these filaments revealed the filaments to be membrane-less and consist of repeats of four SorCS2 dimers with D4 symmetry, stacked with a slight helical twist of approximately 9° (Fig. 6b).

The helix is stabilized by two interfaces. The first corresponds to the interface used in the “head-to-head” dimer-of-dimers arrangement that was found in the SorCS2^DG^ helical assembly and for membrane-bound SorCS2^glyc^ (Fig. 6c). The second corresponds to the “face-to-face” arrangement that was found for membrane-bound SorCS2^glyc^ (Fig. 6d). All SorCS2 dimers in the helical arrangement are in the extended conformation found in the crystal structure of the deglycosylated and unliganded SorCS2 ectodomain (PDB:6FG9) (Leloup et al., 2018). As the His-tagged C-termini of all proteins point at a strong central density in the helix, its formation is suspected to be an artifact arising from interactions between the His-tags and cations (Supplementary fig. 4b). Indeed, addition of Ni^2+^ ions to His-tagged SorCS2 in solution resulted in the formation of filaments of the same dimensions as assessed by negative stain electron microscopy (Supplementary fig. 4c), indicating that His-tag-chelatable cations may have been present in the PGRN sample.

## 3 Discussion

Membranous model systems bridge the gap between structural studies on isolated ectodomains and *in situ* electron cryo-tomography. Two tools to make proteoliposomes for imaging, through a strain-promoted alkyne-azide cycloaddition (SPAAC) and through nickel affinity, were set up and compared.

SPAAC requires site-specific ligation of an azide to a lysine in the LAP2-tag of a protein to allow for covalent coupling to liposomes containing DBCO-PE. The enzymatic addition of the azide to the LAP2-tag is highly specific, as no off-target effects were observed, and allows for straightforward re-purification of azide-ligated protein using size-exclusion chromatography. Coupling is probably not 100% complete, and therefore not every ectodomain is able to attach to the membrane. In tomograms of SPAAC proteoliposomes, proteins are associated with the surface, but liposomes seem inhomogeneously decorated. Liposomes that are decorated, have good surface coverage, but many liposomes are not decorated by protein at all. Whether this heterogeneous protein distribution is due to an inhomogeneous distribution of DBCO-PE lipids over the liposomes will need to be determined.

His-tagged proteins can be directly coupled to NTA(Ni)-containing liposomes without the need for an *in vitro* azide ligation. In our hands, the distribution of His-tagged proteins onto NTA(Ni)-containing liposomes is more homogeneous compared to the alkyne-azide cycloaddition method. One should be aware however, that the His-tag association with DGS-NTA(Ni) is reversible allowing intermixing of proteins on the liposomes. Therefore, NTA(Ni) liposomes are suitable for imaging homophilic and heterophilic interactions in *cis*, and homophilic interactions in *trans*. For heterophilic interactions in *trans* however, the covalent SPAAC coupling is more suitable as intermixing should be prevented to maintain the asymmetry between the protein–membrane assemblies.

Imaging of liposomes by electron cryo-tomography is influenced by lipid composition and liposome size (Nsairat et al., 2022). Liposomes used in this study are made of 95% DOPC and have more fluid membranes, leading to greater deformations and more multilamellar vesicles. Larger liposomes can harbor more proteins on the surface and have flatter surfaces that more closely resemble the plasma membrane. However, the increased sample thickness from larger liposomes also leads to a loss in signal in TEM images, impeding subtomogram averaging. Smaller liposomes will be embedded in thinner ice, leading to better signal-to-noise ratio in TEM images and allowing subtomogram averaging to sub-nanometer resolution of the 220 kDa SorCS2 dimer.

Besides using proteoliposomes, studies of proteins in vesicular environments can also be performed with cell-derived vesicles (Zeev-Ben-Mordehai et al., 2014a; Gonzalez-Magaldi et al., 2025). These contain proteins with their native transmembrane sequence, and a native cellular membrane composition, both factors that may impact on protein organization. Yet in doing so, some of the advantages that come with working with ectodomains are lost. Ectodomains can be purified, ensuring that the imaged samples are homogeneous and of high quality, while cell-derived vesicles may contain proteins other than the protein of interest. On the other hand, some ectodomains may be unstable or prone to aggregation during sample preparation, leading to protein aggregates. In addition, protein-induced clustering of proteoliposomes has an adverse effect on imaging due to the increase of sample thickness and inhomogeneous distribution on the grid. Membrane-conjugated ectodomains also lack their native transmembrane sequence, that may contribute directly to protein interactions or play a role in orienting the ectodomain to the membrane (Moore et al., 2008). The His-tag and azide-ligated LAP2-tags used for protein conjugation to liposomes do not necessarily impose the exact same spacing as the native transmembrane anchoring does. However, ectodomains of type-I and type-II transmembrane proteins are regularly connected to their transmembrane helix by short stalks, i.e. unstructured linkers of a few amino acids, affording them flexibility with regard to the membrane. Such flexibility is replicated in the two coupling methods described in this work.

One of the most challenging parts of subtomogram averaging workflows is the initial localization of particle positions and orientations (Pyle et al., 2022). For the different datasets, we employed three approaches to this problem: template matching and oversampling of surfaces for the SorCS2^DG^ dataset, and manual annotation for the SorCS2/p75^NTR^/NGF dataset.

While recent improvements have increased the reach of template matching for particle localization (Chaillet et al., 2023; Wan et al., 2024; Cruz-León et al., 2024; Chaillet et al., 2025; Majtner et al., 2025), the 220 kDa SorCS2 dimer remains on the lower limit of what's possible (Gubins et al., 2020; Gubins et al., 2022). Consequently, template matching resulted in a high degree of false-positive particles being identified, from 58.8 k initial positions to 13.2 k particles in the final average (Supplementary fig. 2a,c). The same template matching approach on the SorCS2(PGRN) dataset, where SorCS2 molecules on the liposomes are seemingly not organized in clusters, did not lead to successful reconstructions of the SorCS2 dimer. Here, the increased ice thickness and resulting lower contrast may have been a limiting factor. Taken together, it appears that for the relatively small SorCS2 dimer the added context of membrane proximity and *cis* interactions contribute substantially to selecting the true positive positions by classification.

By oversampling the liposome surfaces, *de novo* reconstruction of the SorCS2^DG^ dimer was possible (Supplementary fig. 2a,b). Interestingly, only 23% of the 16.2 k particles in the final set were also identified by template matching, indicating a relatively low recall for both methods, and raising the question of how many more particles remain unidentified in the tomograms.

For the SorCS2(p75^NTR^/NGF) dataset, particle positions were annotated manually, to overcome difficulties with oversampling the more variable membrane shapes. The major advantage of manual picking is that, as long as particles are clearly visible in the denoised tomograms (Buchholz et al., 2019), generally one position is defined per particle. The initial orientation of the particle, perpendicular to the membrane, is retrieved by an initial global alignment to a reference with a membrane and nondescript protein layer. We included this orientation as a prior to restrain subtomogram averaging. From this set of 30,828 manually annotated membrane-associated densities, 952 were refined to a SorCS2 “head-to-head” and 625 to a “face-to-face” dimer-of-dimers. Presumably, not all of the particles on the membranes in this dataset adopt either dimer-of-dimer conformation, but other conformations could not be refined. The extra mass arising from the second dimer probably helps in resolving these arrangements from the heterogeneous pool of particles.

**Fig. 7:**
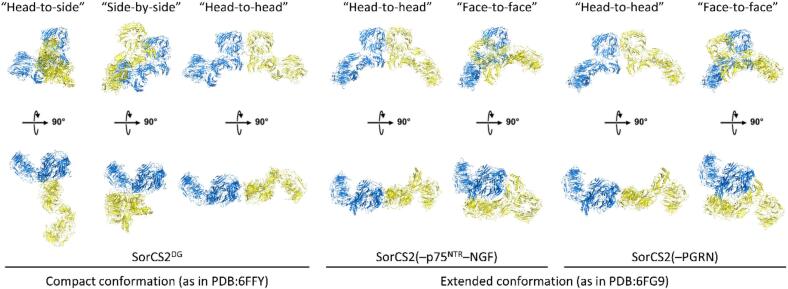
Overview of SorCS2 *cis* interactions. A summary of the four dimer-of-dimer arrangements found in the three tomography datasets. The SorCS2 dimer conformation, compact as in the SorCS2–NGF crystal structure (PDB:6FFY) (Leloup et al., 2018), or extended as in the crystal structure of deglycosylated SorCS2 (PDB:6FG9) (Leloup et al., 2018), is indicated.

Cryo-electron tomography enabled the structural analysis of the SorCS2 ectodomain on membranes. We posed that the added context of the membrane could influence protein conformations, as well as provide a surface for *cis* interactions. Indeed, four modes of self-interaction of SorCS2 dimers to form dimer-of-dimer arrangements are found (Fig. 7). From previous structural work on the ectodomains of SorCS proteins, structural plasticity can be inferred (Leloup et al., 2018; Januliene et al., 2017b; Dong et al., 2022), with the extended conformation found in the crystal structure of SorCS2^DG^ and the compact crystal structure of SorCS2 in complex with NGF, representing two extremes (PDB: 6FG9,6FFY) (Leloup et al., 2018). The conformation of SorCS2^DG^ on the membrane more closely represents that of NGF-bound SorCS2 (PDB: 6FFY), while the conformation found in the SorCS2(p75^NTR^/NGF) sample is more similar to the SorCS2^DG^ crystal structure (PDB: 6FG9). This indicates that glycosylation does not play a direct role in restricting one conformation over the other, but rather that SorCS2 has intrinsic conformational plasticity. The extended conformation of the SorCS2 dimer is not compatible with the “head-to-side” arrangement found in the SorCS2^DG^ tomograms on membranes and in the helical arrangement, as it would clash with the docked dimer. An N-linked glycosylation site on Asn830 is located near the PKD1–10CC-b interface of the “head-to-side” arrangement. Modeling of a glycan on Asn830 indicates that the glycan does not directly clash with the other dimer in the “head-to-side” arrangement, but the proximity of the glycan to the interface might influence this arrangement in fully glycosylated SorCS2 (Supplementary fig. 5a). The “head-to-head” dimer-of-dimers arrangement is found in all three analyzed datasets, and is compatible with compact and extended SorCS2 dimers (Fig. 7).

For the SorCS2 ectodomain, which is located luminally or extracellularly, self-association as seen on membrane tubules and liposome surfaces is associated with negatively curved membranes. This is topologically the opposite of what would be expected for the known physiological roles and localization of SorCS2: during endocytosis, vesicular transport, and in the cisternae of the Golgi network, membranes would largely be positively curved (McMahon and Gallop, 2005). Perhaps inducing negative curvature by *cis*-interactions is a way for unliganded SorCS2 to avoid positively curved membranes and premature endocytosis, a mechanism also proposed for Mucin-1 (Lu et al., 2022). The “head-to-side” self-interaction interface underlying the helical SorCS2^DG^ organization on membrane tubules and found on liposome surfaces, directly competes with the known binding site for (pro)NGF and (pro)BDNF (Leloup et al., 2018). In the SorCS2(p75^NTR^/NGF) sample, one protomer of each SorCS2 dimer has its (pro)neurotrophin binding site obscured by positioning on the membrane. A set of these dimers further form the “face-to-face” arrangement, which also sterically blocks the binding site on the other protomer. This indicates that at high local concentration on the membrane, SorCS2 *cis*-interaction may auto-inhibit neurotrophin binding.

A physiological role for the helical arrangement of SorCS2^DG^ on membranous tubules is not immediately obvious. The protein-crowding-mediated formation of membranous tubules has been well-established in *in vitro* systems (Stachowiak et al., 2010; Yuan et al., 2021), and cell-derived membranes can also form tubules, such as observed for vesicles from cells overexpressing the fusogen EFF-1 (Zeev-Ben-Mordehai et al., 2014b). Mechanistically, the presence of a high number of molecules on the membrane surface results in entropic pressure, which causes membrane bending (Stachowiak et al., 2012; Zeno et al., 2018; Shurer et al., 2019). In the SorCS2^DG^ membranous tubules, however, permissible conformations are instead restricted by specific protein–protein interactions, reducing conformational entropy. As such, they more closely resemble membrane tubules induced by the GTPase Dynamin or BAR domain-containing proteins that form specific helical coats (Frost et al., 2008; Danino et al., 2004; Chappie et al., 2011; Ferguson and De Camilli, 2012; Simunovic et al., 2019).

A recent study identified a SorCS2 missense variant in a family with persistent attention-deficit/hyperactivity disorder (Kaas et al., 2025). The characterization of this Arg702Trp mutant revealed aberrancies in SorCS2 cellular trafficking, most notably its retention in the endoplasmic reticulum. Arginine 702 is localized in the 10CC-b domain and is centrally positioned in the “head-to-side” interface of the PKD1 domain of the other SorCS2 dimer (Supplementary fig. 5b). The interfacing residues on PKD1, Phe824, Ala826, and Tyr828, are hydrophobic/aromatic. Potentially, the Arg702Trp substitution on 10CC-b would increase the hydrophobic/aromatic nature of the interface and thus strengthen SorCS2 *cis* interactions, thereby influencing cellular trafficking. Whether and how the four distinct membrane-stabilized SorCS2 *cis* interactions affect SorCS2 trafficking or SorCS2-mediated cell signaling will, however, need to be established.

## 4 Methods

### 4.1 Protein expression and purification

NGF and the SorCS2 ectodomain used for deglycosylation and imaging of SorCS2^DG^ were expressed and purified as described in Leloup, *et al. (*Leloup et al., *2018**)*. In short, constructs consisting of residues 19–241 of murine NGF (which includes the propeptide) and of residues 117–1072 of murine SorCS2 were expressed in N-acetylglucosaminyltransferase I-deficient (GnTI−) EBNA1-expressing HEK293 cells (HEK293-ES; ImmunoPrecise Antibodies). Medium was harvested and diafiltrated to a buffer of 25 mM 4-(2-hydroxyethyl)-1-piperazineethanesulfonic acid (HEPES) at pH 7.5 and 500 mM NaCl, and purified by tandem nickel-affinity immobilized metal affinity chromatography (IMAC) and size-exclusion chromatography (SEC) to a buffer of 20 mM HEPES pH 7.0 and 150 mM NaCl for NGF and 25 mM 2-(N-morpholino)ethanesulfonic acid (MES) at pH 5.5 and 500 mM NaCl for SorCS2. SDS-PAGE analysis revealed that proNGF had proteolytically been converted to its mature form (Leloup et al., 2018). SorCS2 was subsequently deglycosylated using Endo Hf 1:100 overnight at room temperature.

Constructs spanning residues 20–508 for murine MAG (Pronker et al., 2016), residues 117–1072 for murine SorCS2 (Leloup et al., 2018) and residues 32–252 of murine p75^NTR^ were subcloned into a vector containing an N-terminal cystatin secretion signal peptide, and a C-terminal His_6_ tag or LAP2-His_6_ tag (Puthenveetil et al., 2009). These were then expressed in EBNA1-expressing HEK293 cells (HEK293-E; ImmunoPrecise Antibodies). Medium was harvested after six days of expression and loaded onto a 5 mL HisTrap™ Excel column (Cytiva) after removal of cell material by centrifugation at 3000 *g* for 15 min, and filtering using a 0.2 μm sterile filter (Corning). The column was then connected to an AKTA PURE (FPLC) system (Amersham Biosciences) and washed first with a buffer of 25 mM HEPES at pH 8.0, 500 mM NaCl and 2 mM CaCl2, and then with the same buffer supplemented with 10 mM imidazole. Proteins were eluted in that buffer supplemented with 500 mM imidazole and concentrated using an Amicon® Ultra-4 Centrifugal Filter Unit with a 10 kDa MWCO for p75^NTR^, a 30 kDa MWCO for MAG, and 100 kDa MWCO for SorCS2. Concentrated proteins were further purified by SEC, on a Superdex™ 200 10/300 column (Cytiva) for p75^NTR^ and MAG, and a Superose™ 6 column for SorCS2, in a buffer of 25 mM HEPES at pH 7.5, 150 mM NaCl, and 2 mM CaCl_2_ (hereafter SEC buffer). Purified proteins were collected and concentrated using Amicon® Ultra-15 Centrifugal Filter Unit with the same MWCO used after IMAC elution.

### 4.2 Liposome preparation

Lipid mixtures were prepared by pipetting chloroform solutions of 95% 18:1 PC (DOPC; Avanti) and 5% 18:1 DGS-NTA(Ni) (Avanti) or 5% 18:1 DBCO-PE (Avanti) to a total lipid weight of 2.5 mg in a glass tube. Lipid mixtures were then dried initially under a stream of nitrogen gas at 37 °C, and then overnight in a vacuum desiccator. Dried films were hydrated in 1 mL of SEC buffer at 37 °C, with intermittent vortexing to a total lipid concentration of 2.5 mg/mL. Hydrated lipid solutions were then aliquoted, frozen, and stored in a − 80 °C freezer. When used, aliquots were retrieved from the freezer, thawed, and extruded to form liposomes using a membrane with 100 nm pore size (Avanti).

### 4.3 Azide ligation and click reaction

The ligation of 8-azidooctanoic acid to LAP2-tagged proteins was performed by pipetting the following components, in the following order: 292 μL of SEC buffer, 50 μL of 50 mM Mg(OAc)_2_ in SEC buffer, 50 μL of 10 mM ATP in SEC buffer, 50 μL of 2.5 mM 8-azidooctanoic acid in SEC buffer, 8.3 μL of 120 μM LplA^W37V^, 50 μL of LAP2-tagged protein. This mixture was then incubated while gently shaking overnight at room temperature. Azide-ligated proteins were re-purified by SEC and concentrated as described for the purification. To test azide ligation and off-target effects, azide ligation was repeated with MAG and SorCS2 constructs that contain a His-tag only, i.e., no LAP2-tag. MAG or SorCS2, with or without LAP2-tag, before or after azide ligation, giving eight total combinations, were incubated with 50 μM of DBCO–Alexa Fluor 488 conjugate (Jena Bioscience). After three hours of incubation, Laemmli sample buffer was added, and samples were loaded on SDS-PAGE. The gel was first imaged for fluorescence, then stained using InstantBlue Coomassie stain, and imaged for Coomassie staining.

To test reactivity of DBCO-functionalized liposomes, 2.5 mg/mL 5% DBCO-liposome stocks, at an effective DBCO concentration of 125 μM, were mixed 1:1 with 125 μM, 25 μM, or 0 μM 8-azidooctanoic acid in SEC buffer in a glass bottom 96-well plate. A UV–Vis spectrum from 220 nm to 400 nm was measured every three minutes for 255 min in a CLARIOstar Plus plate reader (BMG Labtech). In order to correct for drifting baselines, signal at the 312 nm DBCO absorption peak was normalized by dividing it by the constant 271 nm signal. A pseudo-first-order kinetic eq. (1) was fit to the absorbance ratio, assuming it to be linearly proportional to the DBCO concentration. As not all DBCO may be accessible on the liposome surfaces, an additional parameter *l* was introduced to describe the asymptotic lower limit of the absorbance ratio.

(1) $$\left[\mathrm{DBCO}\right]={\left[\mathrm{DBCO}\right]}_{0}e^{-\mathrm{kt}}+l\;$$

### 4.4 Electron cryo-tomography sample preparation and data collection

DBCO- or NTA(Ni)-functionalized liposome samples at a concentration of 2.5 mg/mL total lipid concentration were prepared as described above. At 5% functionalized lipids and 50% surface accessibility, this would correspond to approximately 63 μM DBCO or NTA(Ni) groups available. For the MAG samples, 1.3 mg/mL protein was mixed with liposomes in a 1:1 mass ratio. For SorCS2^DG^, this was 1 mg/mL. For SorCS2(PGRN), SorCS2 was mixed with commercially available PGRN (AdipoGen) at 1.3 mg/mL in a 1:1.1 M ratio, and this mixture was then mixed with liposomes in a 1:1 mass ratio. For SorCS2(p75^NTR^/NGF), SorCS2 and p75^NTR^, both at 1.3 mg/mL, were mixed with liposomes in a 1:1:2 mass ratio. Each of these samples were incubated while shaking overnight at room temperature. The samples were then each four-fold diluted to a final lipid concentration of 0.3 mg/mL, as this gave the most consistent results for liposome distribution on the grid. For the SorCS2–p75^NTR^–NGF complex sample, a three times excess of NGF to SorCS2/p75^NTR^ was added at this step, and incubated for an hour. 4 μL of sample was pipetted onto grids, Quantifoil 2/2300 for MAG, and Quantifoil 1.2/1.3300 for all SorCS2 variants. Excess liquid was blotted away, and grids were plunge frozen in liquid ethane using a manual plunging device.

Tilt series of functionalized liposomes with coupled proteins were acquired using SerialEM (Mastronarde, 2005) on a Talos Arctica electron microscope (Thermo Fisher Scientific) at the Utrecht University Electron Microscopy Centre, operating at 200 kV, equipped with a K2 camera (Gatan). Tilt series were collected in a dose symmetric tilt scheme with 3° increments over a total range of −54° to +54° (Table 1). For the SorCS2(p75^NTR^/NGF) sample, tilt series were acquired in parallel using PACE-tomo (Eisenstein et al., 2023).

**Table 1:** Tomography data collection parameters.

Dataset	SorCS2^DG^	SorCS2(PGRN)	SorCS2(p75^NTR^/NGF)
Microscope	TFS Talos Arctica	TFS Talos Arctica	TFS Talos Arctica
Camera	Gatan K2	Gatan K2	Gatan K2
Magnification	79,000×	79,000×	79,000×
Acceleration voltage (kV)	200	200	200
Collection software	SerialEM	SerialEM	SerialEM + PACE-tomo
Tilt range	−54° to 54° (3° increment)	−54° to 54° (3° increment)	−54° to 54° (3° increment)
Images per tilt series	37	37	37
Dose per image (e^−^/Å^2^)	2.72	2.47	2.66
Frames per image	10	12	10
Defocus range (μm)	−1.5 to −3.0	−1.5 to −3.0	−1.5 to −3.0
Pixel size (Å)	1.72	1.72	1.72
No. of tilt series	58	38	232

### 4.5 Data processing of SorCS2^DG^ dataset

Collected frames were motion-corrected using Warp (Tegunov and Cramer, 2019) and the CTF estimated using CTFPLOTTER (Mastronarde, 2024) Tilt series were aligned and four-times binned (i.e., to a pixel size of 6.88 Å), strip-based CTF corrected tomograms were reconstructed using batch processing in IMOD, making use of gold fiducial markers (Mastronarde and Held, 2017).

Particles in the 58 tomograms were selected using two separate approaches: by oversampling of the liposome surfaces, and by template matching. First, the tomograms were imported into Dynamo (Castaño-Díez et al., 2012), and the protein layers on membrane surfaces were defined, using “surface”, “vesicle”, or “filament rings” models, depending on the shape of the liposome, with 50 Å spacing between picked positions for each model, effectively oversampling the surface. The resulting 129 k particles (22 k from “surface” and “vesicle” models, 107 k from “filament rings” models) were imported into RELION-4.0 (Zivanov et al., 2022), along with the tilt series alignments, CTF models, and other metadata. Pseudo-subtomograms were generated and subjected to 3D classification with alignments enabled. In total, four rounds of classification were used to identify as many particles as possible, using an average of the oversampled positions, filtered to 60 Å, as starting reference (Supplementary fig. 2b). After merging classes with observable SorCS2 density and removing overlapping positions, this resulted in 22.7 k particles which were refined to a resolution of 14 Å. The internal C2 symmetry of the SorCS2 dimer was enforced during a subsequent refinement, and overlapping positions were removed again. In order to further improve the map, the dataset was reprocessed from the collected frames in RELION-5.0 (Burt et al., 2024). This allowed use of Bayesian polishing, CTF refinement, extracting the particles as two-dimensional image stacks, and regularization using the Blush algorithm during classification and refinement (Kimanius et al., 2024). These factors combined to reconstruct a map at 11 Å resolution from 16.2 k particles, with marked improvements in visible features. As an alternative, particle positions were identified using template matching with pytom-match-pick (Chaillet et al., 2025). Having indication that the SorCS2^DG^ dimer adopts a more compact conformation in this dataset, a simulated map of the SorCS2 dimer based on the compact conformation found in complex with NGF (PDB:6FFY) was generated to a resolution of 15 Å with a pixel spacing of 6.88 Å (i.e., Nyquist frequency of 13.8 Å) and used as template. Template matching was performed on tomograms with pixel spacing of 6.88 Å and with 7° angular sampling, and corrections for the accumulated dose and CTF were applied. Given that the SorCS2 dimer has a relatively small mass of only 220 kDa, only weak correlation peaks were observed, and strong correlation was also observed to liposome membranes. In order to select for these peaks, an as yet unpublished neural network with a U-Net architecture was trained on refined positions from the oversampling method to select particle positions. This nonetheless resulted in picks outside of the ice layer, and distant from liposome surfaces. These were manually excluded using the ChimeraX plugin ArtiaX (Ermel et al., 2022), going from 58.8 k initial positions to 38.0 k after curation. The particle locations were imported to RELION-4.0, and extracted pseudo-subtomograms were subjected to classification without alignment. Five of the resulting classes, representing 30.8 k particles, resembled the SorCS2 dimer. Four of these had visible density for the membrane and adjacent particles, while one did not have any more density than was present in the template. Including this latter class resulted in more noisy and lower resolution reconstructions, likely due to false positive particle positions (Henderson, 2013), and it was excluded from further processing. The remaining 17.4 k particles were processed similarly to the particles from oversampling, resulting in a final set of 13.2 k particles that refined to 9.6 Å. The slightly better resolution of this set as compared to the oversampled picks, might be a result of lower heterogeneity due to using a template for particle selection.

A SorCS2 dimer model based on the compact conformation found in the NGF-bound crystal structure (PDB:6FFY) (Leloup et al., 2018) was rigid-body real-space refined into the density maps using Phenix (Liebschner et al., 2019). Rigid bodies were defined as: the beta-propeller as residues 131–641, 10CC-a as residues 643–699, 10CC-b as residues 700–776, PKD1 as residues 778–870 PKD2 as residues 874–956, and SoMP as residues 958–1066. Cross-resolution between the refined model and the map was calculated by simulating the model at a resolution of 4 Å, and calculating the Fourier shell correlation with EMAN2 (Tang et al., 2007).

In order to refine the helical structure on the membranous tubules, the “filament rings” models defined previously in Dynamo were converted to regular filament models, with positions defined along the backbone with 50 Å spacing. These 5558 positions were then imported into RELION-4.0, and four-times binned pseudo-subtomograms were extracted and classified with alignments enabled, resulting in one class of 1681 particles with helical density surrounding the membrane tubules. Helical parameters were estimated using the HI3D web-based tool (Sun et al., 2022), and subsequently refined simultaneously with the map to reach a rise of 32.6 Å and a twist of −134.4° for this triple-start helix. Copies of the real-space refined SorCS2 dimer model were placed in the proteinaceous density.

Particles refined from the oversampling and template matching approaches were merged, overlapping particles were removed, and the positions were plotted back to the tomographic frame of reference in ArtiaX. Membranes were segmented using MemBrain-seg (Lamm et al., 2025), and segmentations were manually cleaned using the ChimeraX volume eraser tool. As SorCS2 dimers were found on liposome membranes in “head-to-side” arrangements, the combined set of particles was refined and classified with C2 symmetry relaxation. Particles were subsequently subjected to focused classification with a mask that encompasses only the additionally docked Vps10p domain. This resulted in a set of 7398 particles that refined to a resolution of 11.5 Å.

Nearest-neighbor analysis was performed using the polysome-stats package from Gemmer, *et al. (*Gemmer et al., *2023**).*

### 4.6 Data processing of the SorCS2(p75^NTR^/NGF) dataset

Collected frames of NTA(Ni)-functionalized liposomes incubated with SorCS2-His, p75^NTR^-His, and soluble NGF were imported, motion corrected, and the CTF was estimated in RELION-5.0. Tilt series were aligned using IMOD gold fiducial alignment, and cryo-CARE denoised tomograms were reconstructed using RELION-5.0 implementations of these programs. In total, 30,828 particle positions were manually annotated in 232 tomograms using the built-in Napari-based particle selection tool. Half the dataset, 19,625 positions in 112 tomograms, was first processed. Particles were extracted as four-times binned two-dimensional image stacks and refined using as reference a previous subtomogram average of a membrane with a protein layer, filtered to 60 Å, to pre-align them to the membrane normal. These initial alignments were then used in subsequent classification, by restricting the rotational search through adding the *–sigma_tilt* and *–sigma_psi* flags with a value of 10°. This classification worked best with 75 iterations, and the Blush regularization algorithm enabled. One class of 11,731 particles resembles a particle on a membrane, although it was not possible to assign the density to any of the proteins present in the sample. This class was further classified, again restricting the angular search and with Blush enabled, resulting in a class of 1897 particles that resembled a SorCS2 dimer on the membrane. This class was refined and SorCS2 features, such as the beta-propeller cavity, were clearly visible. The initial refinement to the membrane and classification to a particle on the membrane were repeated for the full dataset, resulting in a set of 20,335 particles. These were then refined with a restrained angular search using the previous map with SorCS2 features as a reference map. The result was classified with restrained alignments, and one resulting class of 10,640 particles was classified once again. This resolved in one class of 952 particles resembling the “head-to-head” dimer-of-dimer arrangement, and one class of 625 particles that additionally contains a dimer in the “face-to-face” arrangement. As SorCS2 dimers in both maps resembled the extended conformation, the previously resolved extended structure of SorCS2 (PDB:6FG9) was rigid-body refined into the density using Phenix, with each protomer defined as a rigid body.

### 4.7 Data processing of the SorCS2(PGRN) dataset and negative stain TEM

Collected frames of NTA(Ni)-functionalized liposomes incubated with SorCS2-His and soluble PGRN were imported, motion corrected, and the CTF was estimated in RELION-5.0. Tilt series were aligned using IMOD gold fiducial alignment, and cryo-CARE denoised tomograms were reconstructed using RELION-5.0 implementations of these programs. Filamentous structures were observed in the tomograms, and 135 particle positions were manually annotated using the built-in Napari-based particle selection tool. Particles were extracted as four-times binned two-dimensional image stacks and classified with alignments enabled. A single class containing all particles showed clear proteinaceous features and was further refined and classified to reveal a non-membranous filamentous structure, with an asymmetric unit consisting of four SorCS2 dimers with D4 symmetry. Since the His-tagged C-termini of this filament pointed towards strong central densities, binding of His-tag chelatable ions, such as Ni^2+^, was suspected. To confirm this, 2 mg/mL SorCS2-His was incubated with or without 50 μM NiSO_4_ overnight. The samples were then diluted fifty-fold, keeping the NiSO_4_ concentration constant, and 4 μL was applied to a carbon-coated copper grid (C400Cu100, EM Resolutions). The drops were incubated for 30 s on the grid, blotted away, washed with water, and stained with 2% uranyl acetate two times. The negatively stained grids were evaluated on a Talos L120C G2 (S)TEM equipped with a Ceta CMOS camera (Thermo Fisher Scientific).

Figures were generated using the IMOD program 3dmod (Kremer et al., 1996), ChimeraX (Pettersen et al., 2021) with the ArtiaX plugin (Ermel et al., 2022), and PyMOL v3.1 (Schrodinger).

## CRediT authorship contribution statement

**J. Wouter Beugelink:** Writing – review & editing, Writing – original draft, Visualization, Validation, Software, Methodology, Investigation, Formal analysis, Data curation. **Bert J.C. Janssen:** Writing – review & editing, Writing – original draft, Validation, Supervision, Resources, Project administration, Funding acquisition, Formal analysis, Conceptualization.

## Declaration of competing interest

The authors declare that they have no known competing financial interests or personal relationships that could have appeared to influence the work reported in this paper.

## Data Availability

The following maps are available in the EMDB: The subtomogram average of SorCS2^DG^ at 10 Å resolution under identifier EMD-58429, the helical arrangement of SorCS2^DG^ on membranous tubules under identifier EMD-58430, the SorCS2^DG^ dimer with an additional docked beta-propeller under identifier EMD-58431, the “head-to-head” and “face-to-face” dimer-of-dimer arrangements found in the SorCS2(p75^NTR^/NGF) sample under identifiers EMD-58432 and EMD-58433, and the non-membranous helical arrangement found in the SorCS2(PGRN) sample under identifier EMD-58434.
