# Supplementary material for: Visualization of membrane-stabilized SorCS2 cis interactions
Source: J Struct Biol X. 2026 Jul 29;14:100155. doi: 10.1016/j.yjsbx.2026.100155 (PMC13448449; doi:10.1016/j.yjsbx.2026.100155)
Supplement: Supplementary file 1 [file mmc1.docx]

**Supplementary figure 1:** Full SDS-PAGE from figure 1C, imaged for Alexa Fluor 488 fluorescence (right), and then stained with Coomassie and imaged again (left).

**Supplementary figure 2:** Overview of SorCS2^DG^ subtomogram data processing workflow. **A)** Slice through a tomogram, with corresponding oversampled points and template matching picks. **B)** Processing of oversampled points. Several rounds of classification with alignments were performed, afterwards merging the resulting classes that resemble the SorCS2 dimer. **C)** Processing of template matching picks. Initial classification of positions without alignments led to five classes that resemble the SorCS2 dimer. Excluding one class (red box outline) that did not contain any density that is not present in the template led to higher resolution and less noisy reconstructions. **D)** Nearest neighbor analysis of the merged particle set. Peaks indicate preference in neighbor positioning in relation to the pictured SorCS2 dimer, consistent with the presence of *cis* interactions. These peaks are more pronounced on tubular liposomes but still present on non-tubular liposomes.

**Supplementary figure 3:** Overview of SorCS2(p75^NTR^/NGF) subtomogram data processing. Manually annotated positions are shown in a slice through a denoised tomogram. Particles were aligned to the membrane, and several rounds of constrained classifications on half the dataset resulted in density that resembles a SorCS2 dimer-of-dimers in the “head-to-head” arrangement. This was used to steer refinement of the full dataset, which resulted in densities that resemble the SorCS2 “head-to-head” (left) and “face-to-face” (right) dimer of dimers.

**Supplementary figure 4:** Ni-ions induce SorCS2 helical filament formation. **A)** Micrograph of a single-particle cryo-EM dataset of the SorCS2–PGRN complex. Aggregates and a filamentous structure are observed. Scale bar is 100 nm. **B)** Central slices through the top and side of the filament. His-tagged C-termini of SorCS2 dimers are indicated with red arrows and point towards a strong central density. Most likely the His-tags interact with chelatable cations. **C)** Negative stain EM micrographs of the same concentration of SorCS2 in absence or presence of 50 µM Ni^2+^ ions. In absence of Ni^2+^, particles are isolated and distributed evenly in the sample. In presence of Ni^2+^, aggregates and filaments form. Scale bars are 100 nm.

**Supplementary figure 5:** Potential impact of SorCS2 N830 glycosylation and R702W mutation on the “head-to-side” dimer-of-dimers arrangement. **A)** Zoomed in view on N830 on PKD1, with a glycan modelled. **B)** Detailed view of the PKD1–10CC-b interface shows its hydrophobic/aromatic nature. The disease-associated R702W mutation might strengthen this interface.
